# Tanshinone IIA regulates human AML cell proliferation, cell cycle, and apoptosis through miR-497-5p/AKT3 axis

**DOI:** 10.1186/s12935-020-01468-5

**Published:** 2020-08-07

**Authors:** Zi-Yuan Nie, Ming-Hui Zhao, Bao-Qian Cheng, Rong-Fang Pan, Tian-Rui Wang, Yan Qin, Xue-Jun Zhang

**Affiliations:** 1 Central Laboratory, Affiliated Hospital of Hebei University, 212 Yuhua East Road, Baoding, 071000 China; 2 Department of Life Science and Green Development, Hebei University, Baoding, 071000 China; 3 Department of Hematology, The Second Hospital of Hebei Medical University, Shijiazhuang, 050000 China; 4 Department of Radiology, Affiliated Hospital of Hebei University, Baoding, 071000 China; 5 Department of Clinical Medicine, Hebei Medical University, Shijiazhuang, 050000 China; 6 Department of Nutrition, The Affiliated Hospital of Qingdao University, Qingdao, 266003 China; 7 Department of Orthopaedic Surgery, The Third Hospital of Hebei Medical University, Shijiazhuang, 050051 China

**Keywords:** Tanshinone IIA, Acute myeloid leukemia, Proliferation, Apoptosis, miR-497-5p, AKT3

## Abstract

**Background:**

The roots of *Salvia miltiorrhiza* are used in traditional Chinese medicine (TCM) and have high medicinal value. Tanshinone IIA (Tan IIA) is the active ingredient of *Salvia miltiorrhiza* which can inhibit the growth of acute leukemia cell lines in vitro, although the mechanism remains unclear.

**Methods:**

CCK-8 assays and BrdU stain were used to evaluate cell proliferation ability. Western blot analysis was used to detect protein expression. miR-497-5p expression level was detected by using qRT-PCR, and Annexin V-FITC/propidium iodide (PI) was used to detect cell apoptosis.

**Results:**

Here we reported that Tan IIA could inhibit cell proliferation, induce cell cycle arrest, and promote cell apoptosis in acute myeloid leukemia (AML) cells. Thus, Tan IIA had the anti-cancer activity in AML cell lines, which was likely mediated by up-regulation of miR-497-5p expression. Our data further showed that in AML cells, the same effects were observed with overexpression of miR-497-5p by a miR-497-5p mimic. We demonstrated that Tan IIA could inhibit the expression of AKT3 by up-regulating the expression of miR-497-5p. We subsequently identified that AKT3 was the direct target of miR-497-5p, and that treatment with Tan IIA obviously reversed the effect of treatment with an miR-497-5p inhibitor under harsh conditions. In turn, PCNA expression was increased and cleaved Caspase-3 was suppressed, which contributed to the growth of AML cells.

**Conclusions:**

Our results showed that Tan IIA could inhibit cell proliferation in AML cells through miR-497-5p-mediated AKT3 downregulation pathway.

## Background

Acute myeloid leukemia (AML), is a highly aggressive bone marrow neoplasm that is characterized by abnormal growth of hematopoietic progenitor cells [1, 2]. Targeted therapy, chemotherapy and bone marrow transplantation are all used in the treatment of patients with AML [3, 4]. However, drug intolerance or recurrence are the common challenges in the treatment of AML [5]. In this regard, understanding of the molecular mechanisms that are responsible for the development and progression of AML, and to identification of novel therapeutic drugs, are imperative to the refractory/relapsed AML patients.

Over the last several decades, there have been several advances in therapeutic drugs for AML [6]. Tanshinone IIA (Tan IIA) is an active ingredient extracted from *Salvia miltiorrhiza*, the roots of which are used in traditional Chinese medicine (TCM) and have high medicinal value. Previous studies have confirmed that Tan IIA has multiple biological functions, including anti-angiogenic activities, anti-oxidative and anti-inflammatory effects, and shows anti-cancer effects in various cancers, including leukemia [7–13]. In our previous research, we found that Tan IIA could induce apoptosis of CML cell lines by down-regulating PKM2 expression. However, whether similar results could be achieved in AML cell lines, and the underlying mechanisms remain unclear. Therefore, the current study aimed to elucidate this anti-leukemic effect and investigate the mechanism of Tan IIA in AML cell lines.

MicroRNAs (miRNAs) are a class of single-stranded noncoding RNAs that are composed of 18–23 nucleotides, which directly bind to the 3′-untranslated regions (3′-UTRs) of target genes to suppress gene expression at the post-transcriptional level [14–16]. Increasing evidence has demonstrated that miRNAs play a crucial regulatory role in the process of tumorigenesis [17–19]. Numerous miRNAs have been reported to be dysregulated in AML [20–22], which implies that miRNAs are potential therapeutic targets for AML. As a tumor suppressor gene, miR-497-5p plays a role in inhibiting tumor cell growth and inducing apoptosis in multiple types of human cancers [23–27]. However, the role of miR-497-5p in AML has not been explored.

In this study, we detected the expression level of miR-497-5p in AML and further explored whether and how Tan IIA exerts an anti-leukemic effect on AML by moderating miR-497-5p and AKT3 expression and thus regulating cell proliferation and apoptosis.

## Results

### Tan IIA inhibits growth and promotes apoptosis of AML cells

**Fig. 1:**
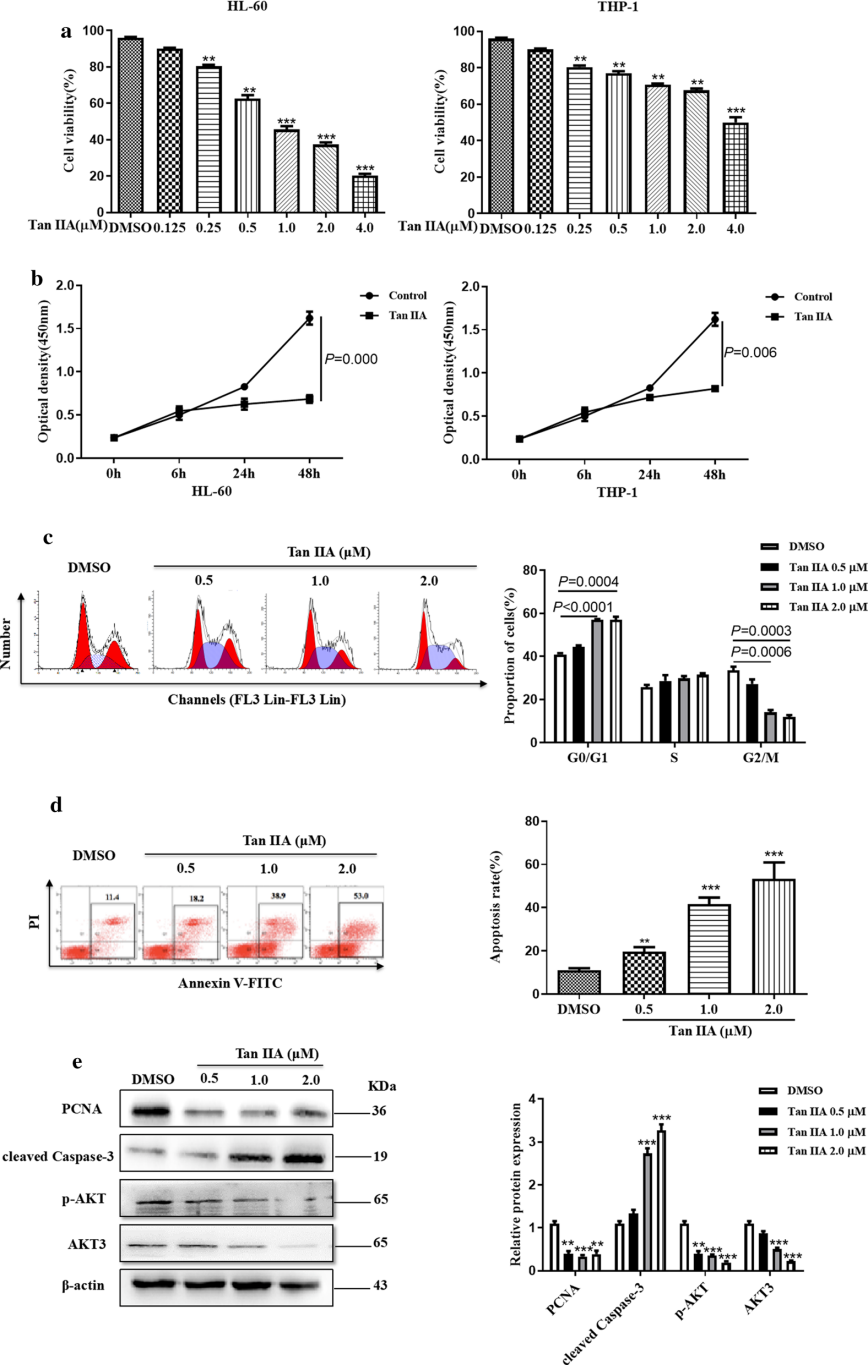
Tan IIA promotes apoptosis and inhibits growth in AML cells. **a** HL-60 and THP-1 cells were treated with various concentrations of Tan IIA for 24 h. At the indicated time, cell viability was analyzed by CCK-8 assay. **P < 0.01, ***P < 0.001 compared to DMSO control. **b** HL-60 and THP-1 cells were treated with Tan IIA for different times. At the appointed time, cell viability was analyzed by CCK-8 assay. **c** HL-60 cells were treated with various concentrations of Tan IIA for 24 h. Cell cycle analysis was conducted by staining the cells with propidium iodide (PI). Right panel shows the number of cells in in different cell cycle phase of three independent experiments. **d** HL-60 cells were treated with (**c**), cell apoptosis analysis was detected with Annexin V and PI staining. Right panel shows the apoptosis rate of three independent experiments. **P < 0.01, ***P < 0.001 compared to DMSO control. **e** HL-60 cells were treated with (**c**), the protein levels of PCNA, cleaved Caspase-3, p-AKT and AKT3 were performed by using western blot analysis. The signal obtained with a β-actin antibody served as loading control. Right panel shows densitometric analysis of three independent experiments. The loading control is β-actin

Tan IIA is the major active component of *Salvia miltiorrhiza*, which has well-recognized clinical anti-tumor effects [28]. Here we sought to determine the effect of Tan IIA on anti-proliferative in AML cell lines (HL-60 and THP-1) by CCK-8 assay. As shown in Fig. 1a, Tan IIA could inhibit the proliferation of AML cells in a dose-dependent manner, and cell proliferation was significantly inhibited by Tan IIA at concentrations of 1.0 μM for HL-60 cells, and 4.0 μM for THP-1 cells. Further, CCK-8 assay was used to evaluate the impact of Tan IIA on the proliferation in AML cells in a time-dependent manner. The results showed that Tan IIA significantly inhibited the proliferation of HL-60 cells at 24 h and THP-1 cells at 48 h (Fig. 1b). HL-60 was found to be the most sensitive cell line to TanIIA. Therefore, HL-60 cell line was consequently selected for the following experiments. Considering the inhibitory effect of Tan IIA on AML cell proliferation, we investigated whether Tan IIA could affect the cell cycle and apoptosis of AML cells. Propidium iodide (PI) staining followed by flow cytometry analysis was used to examine the effect of TanIIA on HL-60 cell cycle progression. As shown in Fig. 1c, absence of TanIIA significantly increased the proportion of HL-60 cells in the G0/G1 phase compared to the DMSO control (Fig. 1c), suggesting that Tan IIA could cause G0/G1 cell cycle arrest in HL-60 cells. Then we examined apoptosis using Annexin V and PI staining and flow cytometry. We found that TanIIA led to a significant increase in the percentage of apoptotic HL-60 cells compared to the control group; the percentage of apoptotic cells increased from 11.4% in the DMSO group to 53% in 2.0 μM TanIIA-treated group (Fig. 1d). We also evaluated the PCNA and cleaved Caspase-3 protein level by using western blot analysis. As shown in Fig. 1e, treatment with Tan IIA decreased the protein levels of PCNA and p-AKT but led to an increase in cleaved Caspase-3 compared with DMSO control. These results suggest that TanIIA can inhibit cell growth, promote apoptosis and prevent cell cycle progression in AML cells.

### Tan IIA induces miR-497-5p expression in AML cells

**Fig. 2:**
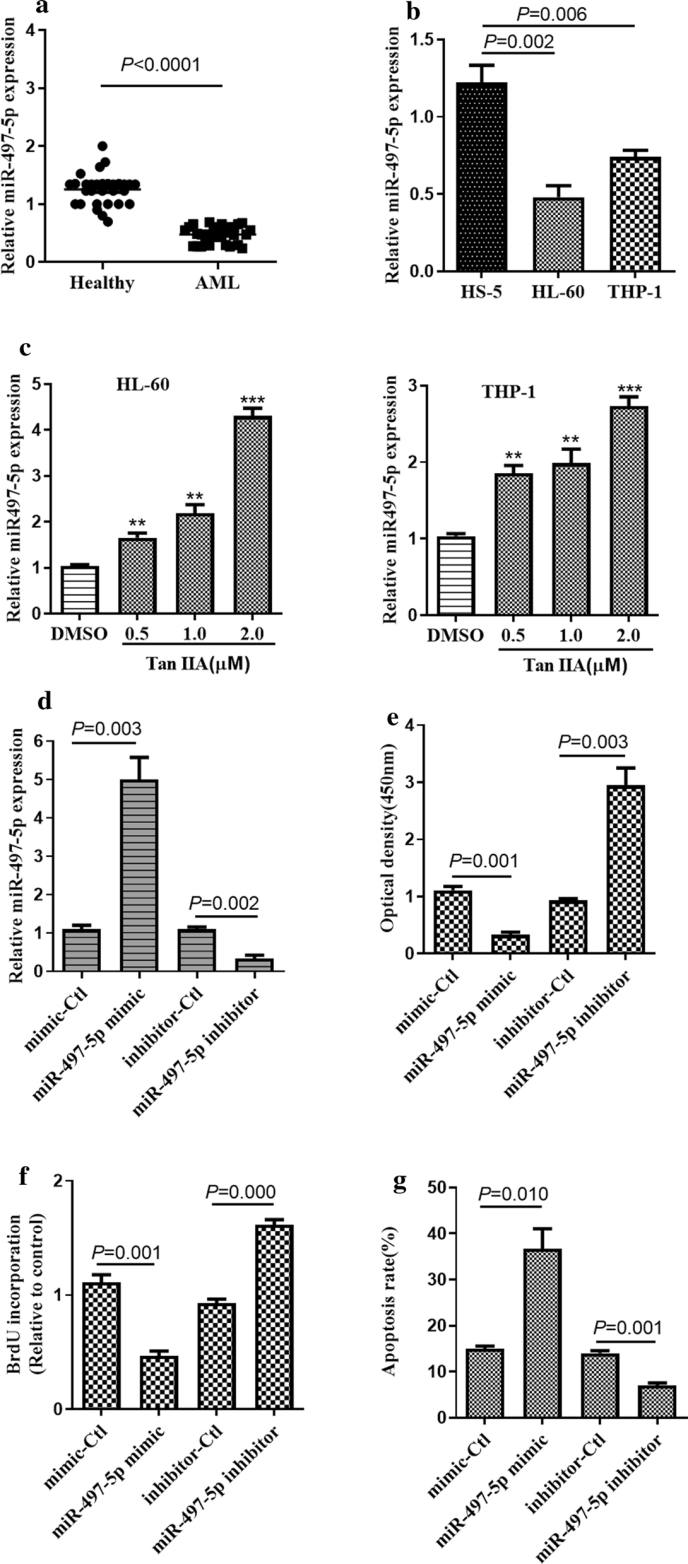
Tan IIA induces miR-497-5p expression in AML cells. **a** RT-qPCR was used to detect the expression level of miR‐497‐5p in the bone marrow cells (BMCs) of AML patients (n = 30) and BMCs of healthy controls (n = 30). **b** RT-qPCR was used to detect the expression level of miR‐497‐5p AML cell lines (HL-60 and THP-1) and normal bone marrow cell line (HS-5). **c** HL-60 and THP-1 cells were treated with various concentrations of Tan IIA for 24 h. The expression level of miR‐497‐5p was examined by RT‐qPCR. **P < 0.01, ***P < 0.01 compared to DMSO control. **d** HL-60 cells were transfected with miR‐497‐5p mimic, miR‐497‐5p inhibitor, or its negative control. The expression level of miR‐497‐5p was examined by RT‐qPCR. **e** HL-60 cells were treated with (**d**), cell proliferation was examined by CCK‐8 assay. **f** HL-60 cells were treated with (**d**), the DNA synthesis ability of AML cells was detected by a bromodeoxyuridine incorporation assay. **g** HL-60 cells were treated as (**d**), cell apoptosis was examined by Annexin V and PI staining

miR-497-5p has been reported to play a tumor suppressor role in numerous cancers. To delineate the expression of miR-497-5p in AML cells, we first detected the expression of miR-497-5p in patients with AML (n = 30) and healthy controls (n = 30). RT-qPCR result revealed that the relative expression of miR-497-5p was significantly downregulated in patients with AML compared to healthy controls (Fig. 2a). Then we tested the expression levels of miR-497-5p in AML cell lines, and the result showed that the expression of miR-497-5p was markedly decreased in both HL-60 and THP-1 compared to that in a normal bone marrow cell line (HS-5) (Fig. 2b). These results indicate that miR‐497‐5p expression is downregulated in AML cell lines, suggesting that the downregulation of miR-497-5p may be related to the development of AML. To verify whether Tan IIA could regulate miR-497-5p expression in AML cells, we treated HL-60 and THP-1 cells with different concentrations of Tan IIA for 24 h. RT-qPCR analysis results showed that treatment of Tan IIA obviously increased the expression levels of miR-497-5p in both HL-60 cells and THP-1 cells (Fig. 2c), although to a greater extent in HL-60 cells. Thereafter, HL-60 cells were chosen to investigate the biological function of miR‐497‐5p on cell proliferation and apoptosis. To do this, miR‐497‐5p was overexpressed or knocked down by transfecting miR‐497‐5p mimics or a miR‐497‐5p inhibitor into HL-60 cells, and expression was confirmed by RT-qPCR analysis. As shown in Fig. 2d, transfection with the miR-497-5p mimic in HL-60 cells significantly increased the expression of miR-497-5p, while transfection with miR-497-5p inhibitor markedly decreased the expression of miR-497-5p in HL-60 cells. Then CCK-8 assay was used to evaluate the impact of miR‐497‐5p on AML cell proliferation. The results showed that overexpression of miR‐497‐5p significantly inhibited cell proliferation, whereas depletion of miR‐497‐5p promoted cell proliferation in HL-60 cells (Fig. 2e). Considering the inhibitory effect of miR‐497‐5p on AML cell proliferation, we investigated whether miR‐497‐5p expression affected AML cell apoptosis using a BrdU incorporation assay. The results showed that the DNA synthesis was remarkably decreased by the transfection of miR‐497‐5p mimic in HL-60 cells whereas increased by the transfection of miR‐497‐5p inhibitor (Fig. 2f). Furthermore, we found that upregulation of miR-497-5p increased while knockdown of miR-497-5p decreased cell apoptosis in HL-60 cells (Fig. 2g). Taken together, these findings suggest that upregulation of miR‐497‐5p by Tan IIA may associated with AML cell proliferation and apoptosis.

### miR-497-5p directly targets the 3′UTR of AKT3 gene

**Fig. 3:**
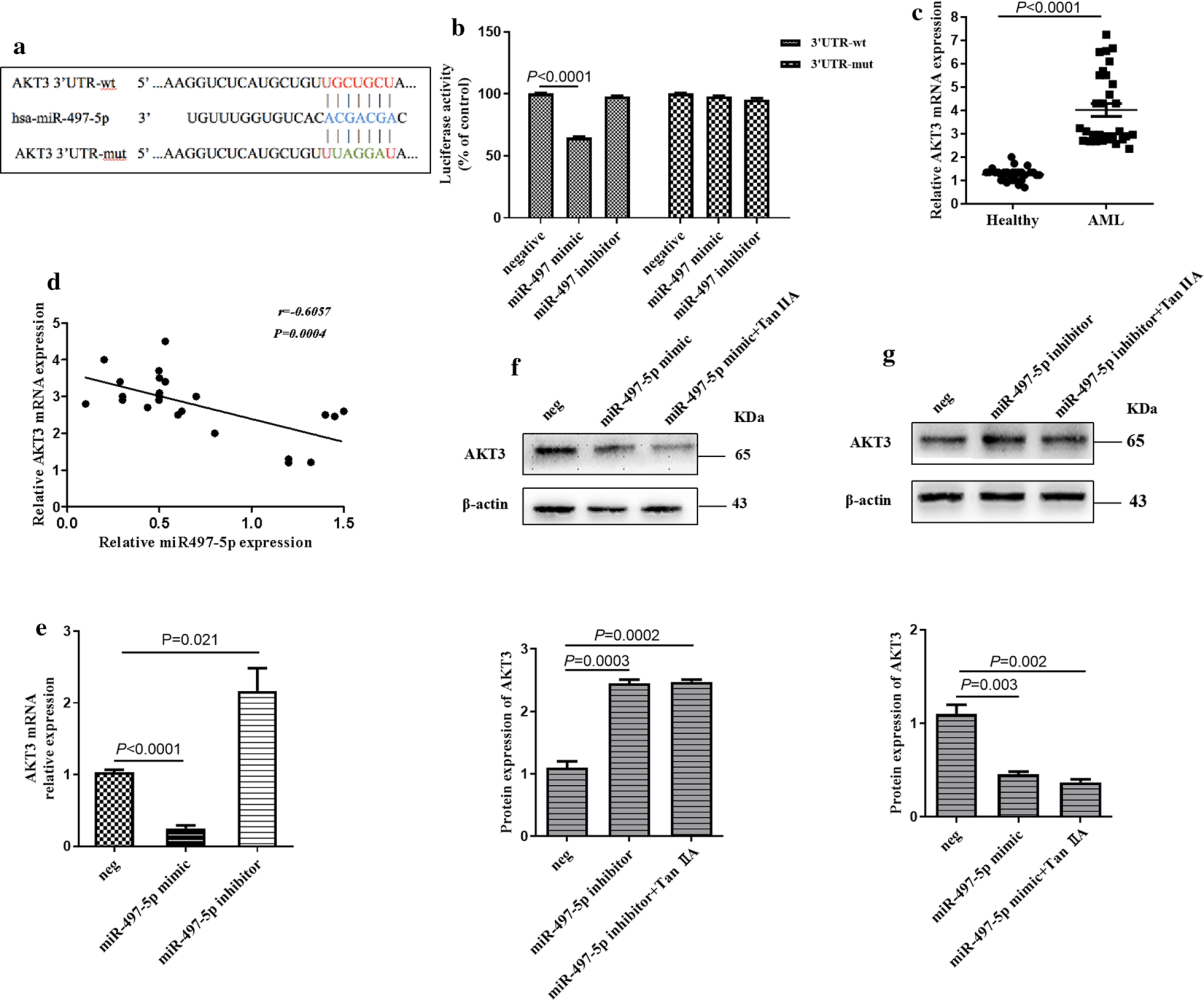
miR-497-5p directly targets the 3′UTR of AKT3. **a** The public miRNA databases, miRDB was scanned to predict targets of miR-497-5p in human. **b** HL-60 cells were co-transfected with miR-497-5p mimic or inhibitor and luciferase reporter plasmid containing wild-type or mutated miR-497-5p-binding site (mut) at AKT3 3′-UTR. Luciferase reporter assay was used to detect luciferase activity. **c** RT-qPCR was used to detect the expression level of AKT3 mRNA in the BMCs of AML patients and in the BMCs of healthy controls. **d** Spearman’s correlation analysis was used to examine the correlation between miR-497-5p and AKT3 mRNA levels in patients with AML. r = − 0.6057, *P *= 0.0004. **e** HL-60 cells were transfected with miR-497-5p mimic or inhibitor, respectively. The expression level of AKT3 in was detected by RT-qPCR. **f** HL-60 cells were transfected with miR-497-5p mimic and then treated with Tan IIA or not. The protein expression level of AKT3 was detected by western blot analysis. **g** HL-60 cells were transfected with miR-497-5p inhibitor and then treated with Tan IIA or not. The protein expression level of AKT3 was examined by western blot analysis

miRNA modulates gene expression by binding to the 3′-UTR of target genes, which subsequently leads to mRNA degradation and/or suppression of translation. The public miRNA database miRDB was scanned to predict targets of miR-497-5p in humans. As shown in Fig. 3a, the 3′-UTR of AKT3 contains a highly conserved binding site for miR-497-5p. Since AKT3 is closely associated with AML progression, AKT3 was selected for further analysis. The luciferase activity assay was first conducted to confirm this hypothesis. The result showed that transfection with miR-497-5p significantly decreased the 3′-UTR wt of AKT3 luciferase activity in HL-60 cell but had no obvious change on the 3′-UTR mut luciferase-plasmid (Fig. 3b), suggesting a direct interaction between miR-497-5p and the 3′-UTR of AKT3. We also examined AKT3 expression in 30 AML patients and 30 healthy controls by using RT-qPCR. The results showed that the mRNA expression of AKT3 was significantly increased in patients with AML compared with health control group (Fig. 3c). Furthermore, the correlation analysis showed that there was an inverse correlation between AKT3 mRNA levels and miR-497-5p in patients with AML (Fig. 3e). We next examined whether miR-497-5p could affect the expression of AKT3 in AML cells. RT-qPCR and western blot analysis showed that overexpression of miR-497-5p decreased AKT3 expression in HL-60 cells at both the mRNA and protein level, while knockdown of miR-497-5p resulted in the increase of AKT3 expression in HL-60 cells at both the mRNA and protein level (Fig. 3e, f). Furthermore, HL-60 cells transfected with miR-497-5p mimics and treated with TanIIA in combination, showed decreased expression of AKT3 compared with overexpression of miR-497-5p alone (Fig. 3e), while HL-60 cells transfected with an miR-497-5p inhibitor and treated with Tan IIA in combination, showed increased expression of AKT3 compared with downregulation of miR-497-5p alone (Fig. 3f). These observations suggest that AKT3 is a direct target of miR-497-5p in AML cells, and that Tan IIA may regulate the expression of AKT3 via miR497-5p.

### Tan IIA inhibits cell growth via blocking AKT3 by miR-497-5p

**Fig. 4:**
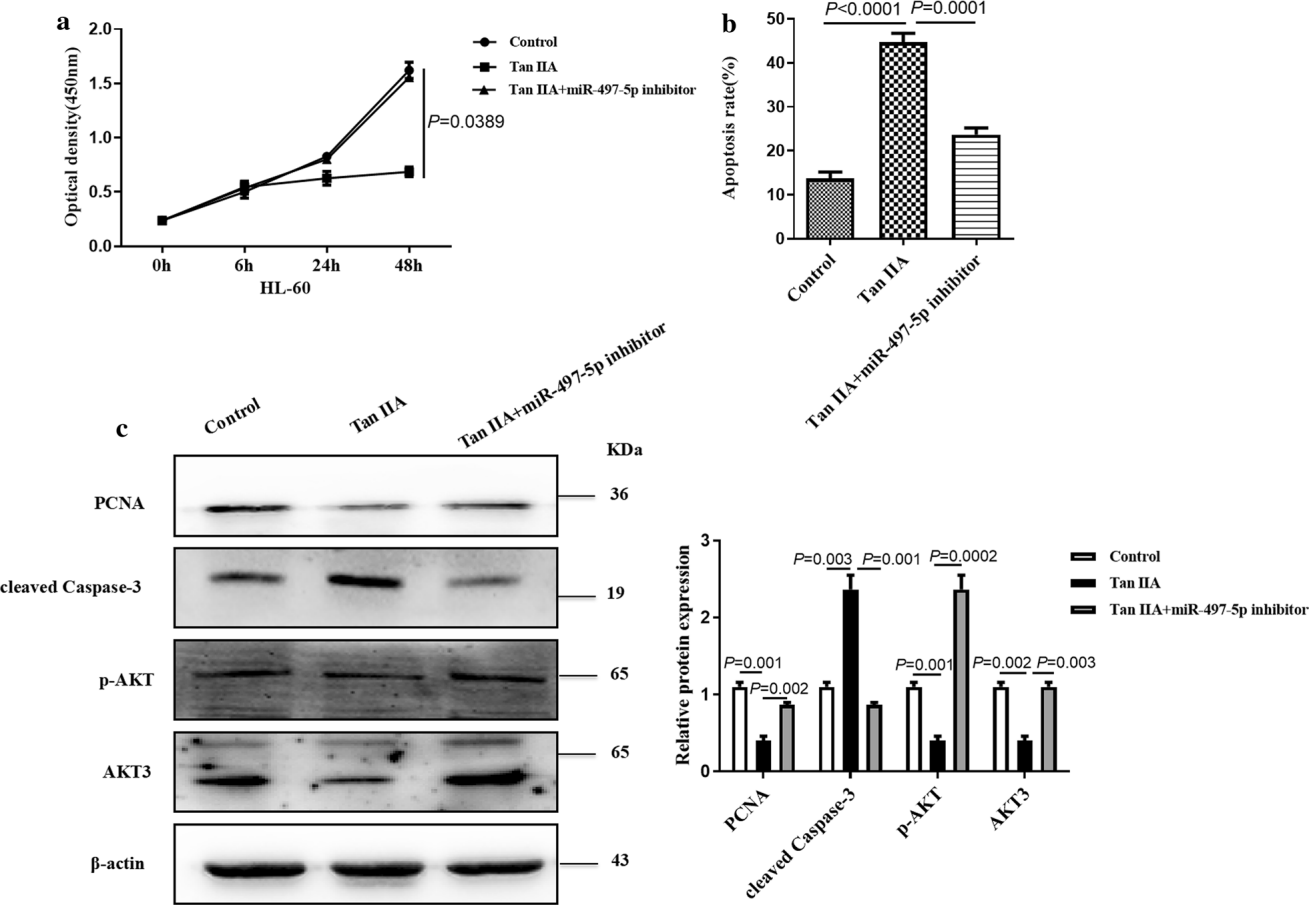
Tan IIA inhibits cell growth via blocking AKT3 by miR497-5p. **a** HL-60 cells were transfected with miR-497-5p inhibitor and then treated with Tan IIA or not. Cell proliferation was examined by the CCK-8 assay. **b** HL-60 cells were treated as (**a**), cell apoptosis was examined by Annexin V and PI staining. **c** HL-60 cells were treated as (**a**), the protein expression levels of PCNA, cleaved Caspase-3, p-AKT and AKT3 was examined by western blot analysis

To investigate the role of miR-497-5p/AKT3 axis on the Tan IIA-mediated inhibition of AML cell growth, HL-60 cells were treated with Tan IIA and then transfected with miR-497-5p inhibitor or not. CCK-8 result showed that Tan IIA could inhibit cell proliferation while this effect was reversed by transfection of miR-497-5p inhibitor (Fig. 4a). Conversely, treatment with Tan IIA and then transfection of miR-497-5p inhibitor in HL-60 cells significantly decreased the cell apoptosis which could be induced by the treatment with Tan IIA alone (Fig. 4b). Western blot results revealed that Tan IIA significantly decreased the protein levels of PCNA and p-AKT in HL-60 cells while the expression of cleaved Casepase-3 was significantly increased. Furthermore, miR-497-5p inhibitor abolished the changes in PCNA, cleaved Caspase 3 and p-AKT protein expression levels induced in response to Tan IIA (Fig. 4c). These findings clearly indicate that miR-497-5p inhibits growth, at least in part, through targeting AKT3 in HL-60 cells.

### Tan IIA suppresses AML xenograft growth in AML in vivo

**Fig. 5:**
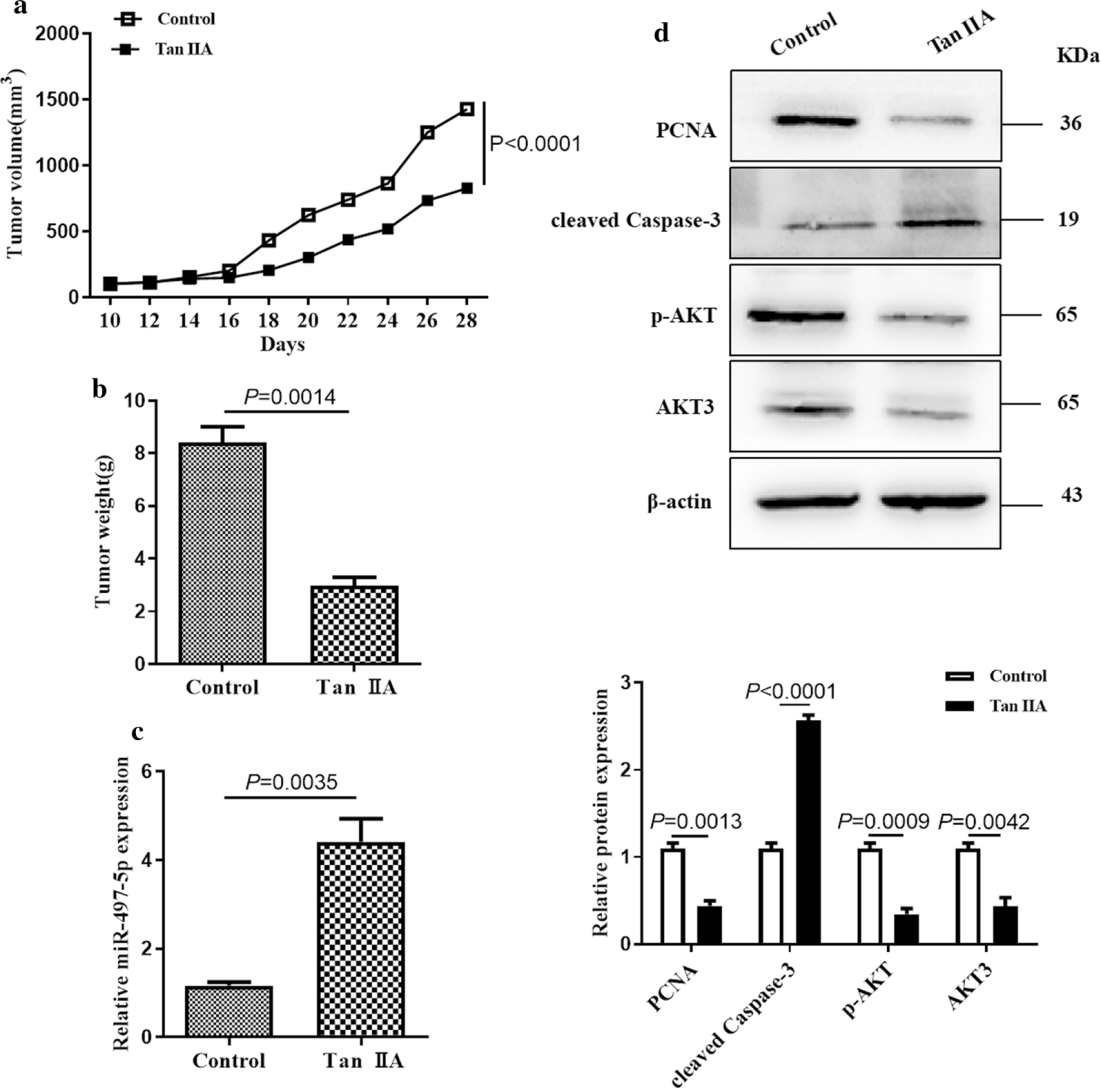
Tan IIA inhibits AML xenograft in vivo. **a** The tumor volume was detected every 2 days for 4 weeks. The tumor volumes were determined with the following formula: Volume (mm^3^) = width^2^ (mm^2^) × length (mm)/2. *P < 0.05 compared to untreated groups. **b** The xenograft tumors formed were excised after 4 weeks. The weights of tumors were detected. **c** RNA was extracted from excised tumors and the expression of miR-497-5p was determined by RT-qPCR. **d** Total proteins were extracted from excised tumors and the levels of PCNA, cleaved Caspase-3, p-AKT and AKT3 were detected by western blot analysis

To determine the effect of Tan IIA on xenograft tumor formation in vivo. HL-60 cells were subcutaneously implanted into nude mice and treated with Tan IIA or PBS. The tumor volume was monitored every 2 days. The result showed that the tumor volume was obviously smaller in Tan IIA group than that in control group (Fig. 5a). In addition, the weight of tumor xenografts in Tan IIA group was significantly decreased (Fig. 5b). The expression level of miR-497-5p in tumor xenografts was analyzed by RT-qPCR to determine whether miR-497-5p upregulation was responsible for the tumor growth suppression in vivo. The results suggested that miR-497-5p expression was increased in the tumor xenografts treated with Tan IIA compared with PBS group (Fig. 5c). Furthermore, the protein levels of PCNA, cleaved Casepase-3 and p-AKT were examined by western blot. The result showed that a significant downregulation in the protein levels of PCNA and p-AKT was observed in the group treated with Tan IIA and upregulation in the protein levels of cleaved Casepase-3 was observe (Fig. 5d). These observations suggest that Tan IIA inhibits the growth of AML cells in vivo via upregulation of miR-497-5p level.

## Discussion

Tanshinone IIA (TanIIA) is a well-known TCM that is extracted from *Salvia miltiorrhiza*. In modern medicine, numbers of research focused on Tan IIA have shown anti-cancer effect in various tumors, including leukemia via inhibiting cell growth and angiogenesis, promoting cell apoptosis and suppressing metastasis and invasion [29]. In our study, CCK-8 assay and flow cytometry revealed that TanIIA exhibits an important anti-tumor effect via inhibition of cell growth, promotion of apoptosis, and retardation of cell cycle progression in vitro in AML cell lines, as well as in an AML xenograft model in vivo. Previous studies found that Tan IIA significantly upregulates the expression of cleaved Caspase-3, while downregulating the expression of PCNA and p-AKT. These results are consistent with those of the current study.

Previous studies have demonstrated that a number of miRNAs are dysregulated during the development of AML. For example, Zhu found that miR‐9 was downregulated in AML patients with poor prognosis [30]. He also reported that upregulated miR-21 was upregulated in AML patients and was associated with poor risk status [31]. Thus inactivation of miR‑21 was considered as, a potential therapeutic option in AML patients. Although an increasing number of studies have shown that Tan IIA has anti-cancer effects via regulation of miRNA expression, to the best of our knowledge, the role of miR-497-5p on the anti-cancer effect of Tan IIA in AML has been unknown [32–34]. The results of the present study indicated that miR‐497‐5p had notably low expression in patients with AML and AML cell lines, and that Tan IIA could inhibit the growth of AML cells growth by upregulating miR-497-5p expression. These findings imply that miRNAs may form a component of a complex regulatory network to mediate the progression and development of AML. Thus, miR-497-5p has the potential to act as a novel therapeutic strategy in the treatment of AML.

We screened the targeted genes of miR-497-5p via the public miRNA database, miRDB, and found that AKT3, serine/threonine protein kinase that plays an important role in cell proliferation, apoptosis, and migration, was a suitable target [35]. In the current study, the interaction between miR-497-5p and AKT3 was determined by the luciferase reporter assay. It has been reported that the AKT signaling pathway is crucial for proliferation in leukemia cells, suggesting that AKT may be a new avenue for leukemia treatment [36, 37]. Recently, a study report that AKT1/2 was overexpressed in K562 cells and promoted cell proliferation and protected the cells from apoptosis [38]. In the present study, our results suggested that AKT signaling pathway was activated in vitro and vivo. More importantly, we confirmed that Tan IIA could inhibit cell proliferation and promote cell apoptosis by moderating miR-497-5p/AKT3 axis.

In conclusion, we first identified that Tan IIA could inhibit AML cells growth by inducing miR-497-5p upregulation. Furthermore, miR-497-5p, targeting to AKT3, inhibited AML cell proliferation, and promoted cell apoptosis. This finding revealed the molecular mechanism of Tan IIA on AML and suggests that miR-497-5p may be of use as a novel specific small molecular target for AML diagnosis and treatment.

## Conclusion

The present study is the first to report that Tan IIA could inhibit acute leukemia cells growth by inducing miR-497-5p up-regulation. miR-497-5p is down-regulated in AML patients and cell lines; whereas, AKT3 is overexpressed. Tan IIA affects the survival of AML cells via the miR-497-5p/AKT3 axis, which may represent a possible target for AML therapy.

## Methods

### Human samples

Bone marrow specimens were obtained from 30 patients with AML and 30 healthy controls from the Second Hospital of Hebei Medical University, between September 2016 and March 2019. The clinical and molecular characteristics of AML patients are provided in the Table 1. A total of 30 cases diagnosed with de novo AML (non-M3) were enrolled. According to the French-America-British (FAB) classification, 4 patients had AML M0, 1 had M1, 7 had M2, 8 had M4, 7 had M5, and 3 had M7. Patients who had been treated with radiotherapy, chemotherapy, or hematopoietic stem cell transplantation before bone marrow aspiration were excluded from this paper. This research was approved by the Ethics Committee of the second affiliated hospital of Hebei medical university. Written informed consent was provided by all patients.

**Table 1:** Patients Characteristics

Characteristics	AML (non-APL) (n = 30)
Age (years),median (range)	51 (19–70)
Male/Female, (n/n)	17/13
WBCs, × 10^9^/L, median (range)	24.9 (2.1–106)
Hb level (g/L)	72 (55–114)
PLT count, × 10^9^/L, median (range)	39 (2–353)

### Cell culture and reagents

Human AML cell lines HL-60 and THP-1 and a normal bone marrow cell line HS-5 were purchased from ATCC and were cultured in DMEM supplemented with 10% (v/v) FBS (Gibco, USA), 100 mg/mL streptomycin, and 100 U/mL penicillin (Sigma-Aldrich, USA) and in a humidified atmosphere conditions containing 5% CO_2_ at 37 °C. Antibodies against PCNA, cleaved Caspase-3, AKT3, AKT, and the secondary antibodies were obtained from Abcam (Abcam, USA). Tanshinone IIA was obtained from MCE (MedChemExpress, USA), and was dissolved in DMSO to make 10 mM stock solution.

### Oligonucleotide design and synthesis

The miR-497-5p mimic and miR-497-5p inhibitor oligonucleotides were designed and chemically synthesized by GenePharma. (GenePharma, China). Cells were seeded at a density of 5 × 10^5^ cells/well. The miRNA mimics and miRNA inhibitors were transfected into cells with Lipofectamine^®^ 2000 (Invitrogen, USA) according to the manufacturer’s protocols. After incubation for assigned time points, transfected cells were collected and used in the following experiments.

### Reverse-transcription quantitative polymerase chain reaction (RT-qPCR)

Trizol reagent (Invitrogen, USA) was used to extract total RNA isolated from both tissue samples or cultured cells using in accordance with the manufacturer’s instructions, and then RNA was reverse transcribed into cDNA using TaqMan MicroRNA RT kit (Thermo Fisher Scientific, USA). The expression of miR-497-5p was detected by using TaqMan MicroRNA Assay Kits (ABI, USA) and the expression of AKT3 was determined using SYGR green real-time PCR (TAKARA, Japan). All the PCR assays were performed with the 7500 PCR Assay System (Grand Island, USA). U6 and GAPDH were used as internal controls. Data were analyzed using the 2^−ΔΔCt^ method, to analyze the relative gene expression. The primers used in this study were as the follows:

miR-497-5p:

Forward: 5′-CCTTCAGCAGCACACTGTGG-3′,

AKT3:

Forward: 5′-GAGTACCTGGCACCAGAGGT-3′,

Reverse: 5′-AGAAAGGCAACCTTCCACAC-3′;

U6:

Forward: 5′-CTCGCTTCGGCAGCACA-3′,

GAPDH:

Forward: 5′-CCCATCACCATCTTCCAGGAG-3′,

Reverse: 5′-GTTGTCATGGATGACCTTGGC-3′;

### Cell counting kit-8 (CCK-8) assay

CCK-8 assay was used to detect the effect of Tan IIA, miR-497-5p mimic or miR-497-5p inhibitor transfection, or the combination on the proliferation of AML cells. In brief, the transfected cells were seeded in 96-well plates at a density of 5 × 10^4^ cells/well in 200 µl culture medium. At appointed time, 10 µl of CCK-8 assay solution (Dojindo Molecular Technologies, Japan) was added to each well, and incubation at 37 °C with 5% CO_2_ for 2 h, the optical density at 450 nm wavelength was selected for the measurement using an ELx808 absorbance reader (BioTek Instruments, USA).

### BrdU incorporation assay

Bromodeoxyuridine (BrdU) incorporation assay (Abcam, USA) performed to detected DNA synthesis ability of AML cells according to the manufacturer’s instructions. In brief, cells were seeded into 6‐well plates and treated with the stimulus for forty-eight hours. BrdU solution (10 μM) was incubated for 2 h and washed with PBS. Cells were fixed with paraformaldehyde for 30 min, followed with Triton X-100 for 15 min. After, cells were treated with BrdU detection antibody (1:1000, ThermoFisher, China) at 4 °C for overnight. Cells were washed with ice-cold PBS for three times. Secondary antibodies which were incubated for 2 h. DAPI was treated for staining of cell nuclear. At last, cells were examined by fluorescence microscopy (Olympus, Japan) with the Image-Pro Plus software (Olympus). Each experiment was performed three times.

### Flow cytometry analysis of cell cycle and apoptosis

The apoptosis rates were detected by flow cytometry (Beckman Coulter, USA) using the Annexin V-FITC/PI kit (Roche, Germany). Briefly, the transfected cells were washed with ice-cold PBS for three times, centrifugated at 200*g* at 4 °C for 5 min, and resuspended in 500 µl binding buffer. Then 5 µl of AnnexinV-FITC and 5 µl of propidium iodide were added into cells for 30 min at room temperature in the dark. A flow cytometer was used to measure the number of apoptotic cells.

The cells were harvested after transfection for 24 h, and washed with ice-cold PBS for three times (Gibco, USA), then fixed with 70% ethanol at 4 °C for at least 2 h. Cells were incubated with 50 µl of RNaseA to degrade endogenous RNA at room temperature for 30 min. Cells were centrifugated at 200*g* at 4 °C for 5 min, followed by the addition of 25 µl of propidium iodide solution and 425 µl of cell staining buffer (both from BioLegend, USA). Cell cycle status was detected by flow cytometer (Beckman Coulter, USA).

### Luciferase reporter assay

Luciferase reporter plasmids such as pmiR-AKT3-3′-UTR wt and pmiR-AKT3-3′-UTR mut were designed and purchased from GenePharma. Cells were seeded in 24-well plates at a concentration of 4 × 10^5^ cells/well. miR-NC or miR-497-5p mimics was co-transfected with pmiR-AKT3-3′-UTR wt or pmiR-IGF-1R-3′-UTR mut into cells via Lipofectamine 2000, according to the manufacturer’s protocols. Luciferase activity was detected using a Dual-Luciferase Reporter Assay System (Promega Corp, USA).

### Protein extraction and western blot

The extracted cell total protein was loaded, separated by 10% SDS-PAGE, and the proteins were transferred onto polyvinylidene fluoride (PVDF) membranes. The membranes were blocked with TBST solution containing 5% nonfat milk at room temperature for 1 h. The membranes were incubated overnight at 4 °C with the following primary antibodies: rabbit anti-human monoclonal PCNA antibody (ab92552; 1:1000 dilution; Abcam, UK), rabbit anti-human monoclonal cleaced casepase-3 antibody (ab2302; 1:1000 dilution; Abcam, UK), mouse anti-human monoclonal antibody to phosphorylated protein kinase B (p-Akt; sc-81433; 1:1000 dilution; Santa Cruz Biotechnology, USA), mouse anti-human monoclonal Akt antibody (sc-56878; 1:1000 dilution; Santa Cruz Biotechnology, USA), and rabbit anti-human monoclonal β-actin antibody (ab179467; 1:1000 dilution; Santa Cruz Biotechnology, USA).The membranes was washed by TTBS for 5 times, and then followed by HRP-linked secondary antibodies (ab6721 and ab6789; 1:1000 dilution; Abcam, USA) for 2 h at room temperature, and the protein signals were detected using an enhanced chemiluminescence reagent (Bio-Rad Laboratories, USA).

### Xenograft tumor experiment

BALB/c nude mice (4–6 weeks) were purchased from the Hebei Medical University Animal Center (Shi Jiazhuang, P.R. China). Collecting HL-60 cells which were in logarithmic growth phase and subcutaneously administered into the hind flanks of nude mice. Tanshinone IIA or PBS injection was given after 1 week. And the mice were observed every day. The width and length of tumor xenografts were examined every 2 days using a vernier caliper. The tumor volumes were analyzed using the equation: tumor volume (mm^3^) = width^2^ (mm^2^) × length (mm)/2. Tumor xenografts were excised from the mice that were sacrificed after 28 days after implantation of cells. Tumor xenografts were collected and used in the following experiments.

### Statistical analysis

All data are represented as mean ± SEM from at least three independent experiments. The two groups data was analyzed using Student’s *t* test. One-way ANOVA was used to compare the differences between multiple groups. Spearman’s correlation analysis was carried out to determine the correlation between miR-497-5p and AKT3 mRNA levels in patients with AML. All statistical analyses were performed using GraphPad software. The P value < 0.05 was indicated statistically significant difference.

## Data Availability

All data generated or analyzed during this study are included in this published article.

## Abbreviations

Tan IIA: Tanshinone IIA
AML: Acute myeloid leukemia
PI3K: Phosphoinositide 3-kinases
AKT3: RAC-gamma serine/threonine-protein kinase
miRNA: microRNA
RT-qPCR: Reverse transcription-quantitative polymerase chain reaction
